# Identification of Flavonoids in the Xylem of *Castanopsis hystrix* and Their Content Variation Patterns During Heartwood Formation

**DOI:** 10.3390/metabo16090655

**Published:** 2026-09-08

**Authors:** Ruoke Ma, Liuming Wei, Pingchuan Zhu, Heng Liu, Boling Liu, Jianhua Qi

**Affiliations:** 1 School of Life Sciences, Qufu Normal University, Qufu 273165, China; maruoke@qfnu.edu.cn; 2 Shandong Key Laboratory of Wetland Ecology and Biodiversity Conservation in the Lower Yellow River, Qufu 273165, China; 3 Guangxi Zhuang Autonomous Region Forestry Research Institute, Nanning 530002, China; weiliuming956@163.com; 4 State Key Laboratory of Conservation and Utilization of Subtropical Agro-Bioresources, Guangxi University, Nanning 530004, China; gchuan12@163.com; 5 Experimental Center of Tropical Forestry, Chinese Academy of Forestry, Pingxiang 532600, China; lljheng@126.com; 6 Heze Forestry Technical Service Center, Heze 274000, China

**Keywords:** *Castanopsis hystrix*, heartwood formation, flavonoids

## Abstract

Background: Flavonoid secondary metabolites accumulate in xylem during heartwood formation and critically influence wood color and economic value. However, the distribution patterns of individual flavonoids across sapwood, transition zone, and heartwood remain poorly characterized in *Castanopsis hystrix*, limiting mechanistic understanding of heartwood formation and rational resource utilization. Methods: Sapwood, transition zone, and heartwood samples were collected and analyzed using ultra-performance liquid chromatography coupled with Q-Exactive Orbitrap mass spectrometry (UPLC-QE-MS) for qualitative and quantitative flavonoid determination. Multivariate statistical approaches, including hierarchical clustering, principal component analysis (PCA), and orthogonal partial least-squares discriminant analysis (OPLS-DA), were applied to compare regional metabolic profiles and to identify characteristic differential markers. Results: A total of 26 flavonoids were identified, covering flavones, flavonols, flavanones, dihydroflavonols, and flavanols. Quantitative and cluster analyses revealed distinct region-preferential accumulation: dihydrorobinetin, dihydromyricetin, morin, and kaempferol were highly enriched in heartwood; fustin, taxifolin, and cyanidin predominated in the transition zone, whereas afzelin and astilbin were more abundant in sapwood. PCA and OPLS-DA further selected four characteristic markers, namely dihydromyricetin, dihydrorobinetin, kaempferol and (−)-epicatechin, that effectively discriminated the three regions. Conclusions: This study establishes a clear radial gradient of flavonoid accumulation in *C. hystrix* xylem, demonstrating selective enrichment of specific metabolites from sapwood to heartwood. These findings provide essential data for deciphering the biochemical mechanisms of heartwood formation and offer a metabolic basis for quality assessment and high-value utilization of this timber species.

## 1. Introduction

*Castanopsis hystrix*, an evergreen arboreal species of the genus *Castanopsis* (Fagaceae), is a precious timber tree endemic to the subtropical region of South China, with a natural distribution in Fujian, Hunan, Guangdong, Hainan, and Guangxi provinces [1,2,3]. The wood of this species is characterized by dense structure, high hardness, and strong decay resistance, conferring significant economic value for high-grade furniture, decorative materials, and premium wood products. Notably, the heartwood of *C. hystrix* exhibits a reddish-brown to deep reddish-brown color, with clear and attractive grain and fine texture, and is frequently utilized as a substitute for rosewood in the manufacture of high-end furniture [4,5]. The proportion and quality of heartwood formation directly determine the commercial value and industrial profitability of the timber [6]; however, the intrinsic mechanisms underlying heartwood formation in *C. hystrix* remain poorly understood, which hinders the targeted cultivation of high-quality heartwood in planted forests and its efficient utilization.

The secondary xylem of trees can be radially divided into sapwood (SW), transition zone (TZ), and heartwood (HW) based on differences in cellular physiological functions and chemical composition [7,8]. Heartwood is derived from sapwood through physiological and metabolic transformation in the transition zone, during which xylem parenchyma cells gradually lose viability and their stored starch and other nutrients are converted into various secondary metabolites that subsequently accumulate in tissues such as vessels and wood fibers [9,10]. Extensive studies have demonstrated that the characteristic color of heartwood is primarily regulated by the types and contents of secondary metabolites, especially flavonoids [11]. For instance, variations in the composition and content of flavonoids in the heartwood of *Dalbergia odorifera* directly determine the intensity of its heartwood color [12]; similarly, color differences between heartwood and sapwood in Chinese fir (*Cunninghamia lanceolata*) and *Taxus chinensis* are closely associated with the accumulation of flavonoid secondary metabolites [13,14]. Moreover, flavonoids often possess antimicrobial properties, which not only enhance aesthetic quality but also improve the decay resistance of wood [15,16,17], thereby increasing its utilization value. Given that flavonoids determine wood color, durability, and commercial value, clarifying their composition and radial distribution in the xylem is essential for understanding heartwood formation and for identifying metabolic markers to support quality assessment and utilization of plantation wood. Therefore, systematic identification of flavonoids in the xylem and elucidation of their distribution patterns along the radial gradient from sapwood through the transition zone to heartwood are essential prerequisites and key entry points for understanding the mechanisms of heartwood formation and achieving efficient wood utilization. To date, however, the flavonoid profile and its spatial distribution across the three xylem regions in *C. hystrix* have not been reported, limiting our understanding of heartwood formation and the efficient utilization of this wood species.

In view of this, the present study used 10-year-old plantation-grown *C. hystrix* wood as material and employed ultra-performance liquid chromatography coupled with Q-Exactive Orbitrap mass spectrometry (UPLC-QE-MS), combined with standard compound comparison and MS/MS fragmentation information, to systematically identify flavonoids in xylem extracts and to analyze their content differences and accumulation patterns across different regions. The specific objectives were: (1) to clarify the flavonoid profile in the xylem of *C. hystrix*; (2) to reveal the radial distribution characteristics of individual flavonoids in sapwood, transition zone, and heartwood; and (3) to screen for differential flavonoid markers that can distinguish the three regions using multivariate statistical models. The findings will provide direct evidence for elucidating the chemical regulatory mechanisms of heartwood formation in *C. hystrix* and will also lay a theoretical foundation and data basis for the targeted extraction and high-value utilization of bioactive flavonoid components from the wood.

## 2. Materials and Methods

### 2.1. Plant Material and Chemical Reagents

The *C. hystrix* samples were collected from the Fubo Experimental Station of the Tropical Forestry Experimental Center, Chinese Academy of Forestry (106.8589° E, 22.0457° N), located in Shangshi Town, Pingxiang City, Chongzuo Prefecture, Guangxi Zhuang Autonomous Region, China. All plant materials have been formally identified by Professor Yunlin Fu (College of Forestry, Guangxi University). Three 10-year-old trees with similar growth vigor were selected. Wood cores were extracted at breast height using an increment borer, immediately immersed in liquid nitrogen, transported to the laboratory on dry ice, and stored at −80 °C until further processing.

### 2.2. Preparation of Standard Solutions

Reference standards of pinocembrin, butin, fustin, (+)-catechin, (−)-epicatechin, morin, dihydrorobinetin, myricetin, dihydromyricetin, and diosmetin (purity ≥ 98%; Shanghai Yuanye Bio-Technology Co., Ltd., Shanghai, China) were separately dissolved in methanol to prepare stock solutions at graded concentrations. All stock solutions were stored at 4 °C. Quantification was carried out using the external standard method. Calibration curves were constructed for each compound by plotting peak area against concentration.

### 2.3. Preparation of Sample Solutions

The cryopreserved *C. hystrix* samples were freeze-dried under vacuum for 24 h and then dissected according to the anatomical structure into sapwood (yellowish-white to yellowish-brown), transition zone (the 5-mm radial width at the color boundary), and heartwood (reddish-brown to deep reddish-brown) with three biological replicates obtained per tissue zone (*n* = 3 per zone). From each region, 0.1 g of sample was accurately weighed and extracted with 1 mL of 80% aqueous methanol (*v*/*v*), to which L-2-chlorophenylalanine was added as an internal standard at a final concentration of 1 ppm (1 mg/L) to monitor instrument stability and data reproducibility during the analytical sequence. The mixture was vortexed and allowed to stand at 4 °C for 24 h. During the extraction, ultrasonic treatment was performed twice in an ice-water bath, each for 30 min. After extraction, the mixture was centrifuged at 13,000 rpm for 15 min at 4 °C, and the supernatant was collected as the sample solution for analysis.

### 2.4. UPLC-QE-MS Conditions

Chromatographic separation was carried out on an ACQUITY UPLC BEH C18 column (1.7 μm, 2.1 mm × 100 mm) using a mobile phase consisting of 0.1% formic acid in water (A) and HPLC-grade methanol (B). The gradient elution program was set as follows: 0–15 min, 5–80% B; 15.0–18.0 min, 80–100% B; 18.0–18.1 min, 100–5% B; and 18.1–21 min, hold at 5% B. The flow rate was 0.3 mL/min, the column temperature was maintained at 30 °C, and the injection volume was 1 μL.

Mass spectrometric detection was carried out on a Q-Exactive Orbitrap mass spectrometer (Thermo Scientific, Waltham, MA, USA) equipped with a heated electrospray ionization (HESI) source operating in positive ion mode. The source temperature was set at 300 °C, the capillary temperature at 320 °C, the sheath gas pressure at 206 kPa, and the auxiliary gas flow at 69 kPa. The spray voltage was 3.0 kV. Data acquisition was carried out in full-scan mode (Full MS) combined with data-dependent MS/MS (Full MS/dd-MS^2^) over a mass range of *m*/*z* 100–1000. Collision-induced dissociation energies were applied at three collision energy ramp levels: 30, 40, and 80 V.

### 2.5. Data Analysis

Compound identification was performed using the XIC Manager module of Compound Discoverer 3.3 software, based on accurate molecular mass, retention time matching with reference standards, mzCloud mass spectral database matching, characteristic MS/MS fragment ions, and comparison with the previously reported literature. The relative peak areas of the 26 identified flavonoids in the sapwood, transition zone, and heartwood were extracted and imported into SIMCA-P 14.1 software for unsupervised principal component analysis (PCA) and supervised orthogonal partial least-squares discriminant analysis (OPLS-DA).

## 3. Results and Discussion

### 3.1. Identification of Flavonoid Components in Xylem Extracts

**Figure 1:**
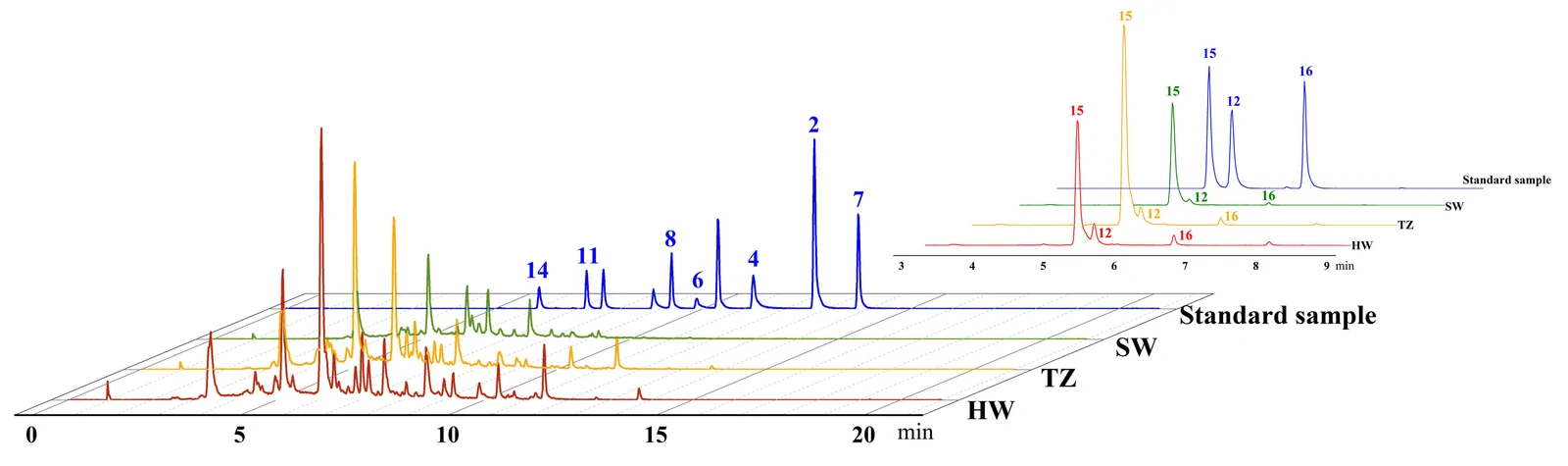
Extracted ion chromatograms (XICs) of flavonoids in *Castanopsis hystrix* xylem based on full-scan UPLC–QE–MS data acquired in positive ion mode. The small image in the upper right corner is a detailed view of the peaks after deconvolution. Diosmetin (2), quercetin (4), myricetin (6), pinocembrin (7), butin (8), fustin (11), dihydrorobinetin (12), dihydromyricetin (14), (+)-Catechin (15), (−)-Epicatechin (16).

The chemical constituents of the sapwood, transition zone, and heartwood of *C. hystrix* were analyzed. A total of 26 flavonoids were identified, all of which are reported for the first time in the wood of this species, and mainly include flavones, flavonols, flavanols, and other subclasses. The total ion chromatograms of the xylem extracts from the heartwood, transition zone, and sapwood are shown in Figure S1. To facilitate visualization of the identified flavonoids, extracted ion chromatograms (XICs) were generated based on the accurate masses of the detected compounds (Figure 1). According to the confidence levels for metabolite identification proposed by the Metabolomics Standards Initiative (MSI) [18], ten compounds—namely pinocembrin, butin, (−)-fustin, (+)-catechin, (−)-epicatechin, morin, dihydrorobinetin, myricetin, dihydromyricetin, and diosmetin—were assigned to Level 1 (i.e., confirmed identification) based on the comparison of their retention times and MS/MS fragmentation patterns with those of authentic standards analyzed under identical experimental conditions. The remaining sixteen compounds were classified as Level 2 (i.e., putatively annotated compounds) based on accurate mass measurements, tandem MS fragmentation behavior, matching with the mzCloud spectral database, and literature reports, in the absence of authentic standard verification. The retention times (RT), mass errors (ppm), and major MS/MS fragment ions for the 26 compounds are summarized in Table 1.

#### 3.1.1. Flavones

**Figure 2:**
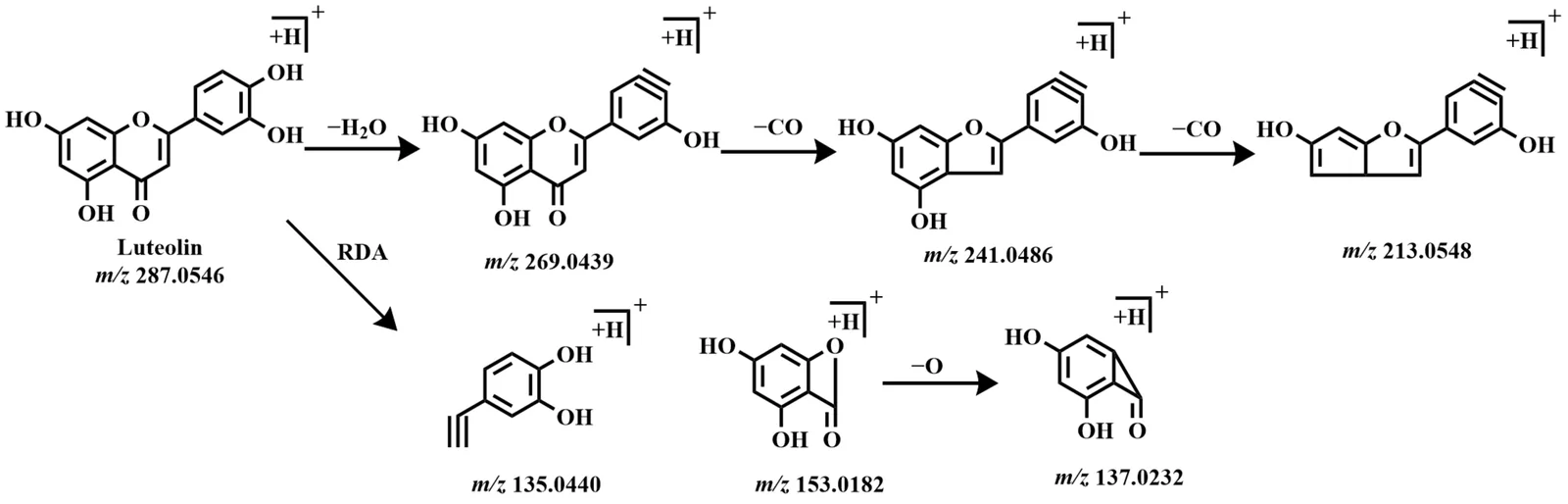
Chemical structure and fragmentation pathways of luteolin.

Compound **1** exhibited a protonated molecular ion [M+H]^+^ at *m*/*z* 287.0546, corresponding to the molecular formula C_15_H_10_O_6_. In the positive-ion mode, the precursor ion sequentially lost one molecule of H_2_O to give a fragment ion at *m*/*z* 269.0439, followed by successive losses of CO to yield ions at *m*/*z* 241.0486 and *m*/*z* 213.0548. The retro-Diels–Alder (RDA) fragmentation of the C-ring generated characteristic ions at *m*/*z* 153.0182 [A_1_,_3_]^+^ and *m*/*z* 135.0440 [B_1_,_3_]^+^. Based on these fragmentation patterns in combination with mass spectral database matching, compound **1** was tentatively identified as luteolin, and its proposed fragmentation pathway is illustrated in Figure 2. Compound **2** was identified as diosmetin by comparison of its retention time and MS/MS fragment ions with those of an authentic standard.

#### 3.1.2. Flavonols

**Figure 3:**
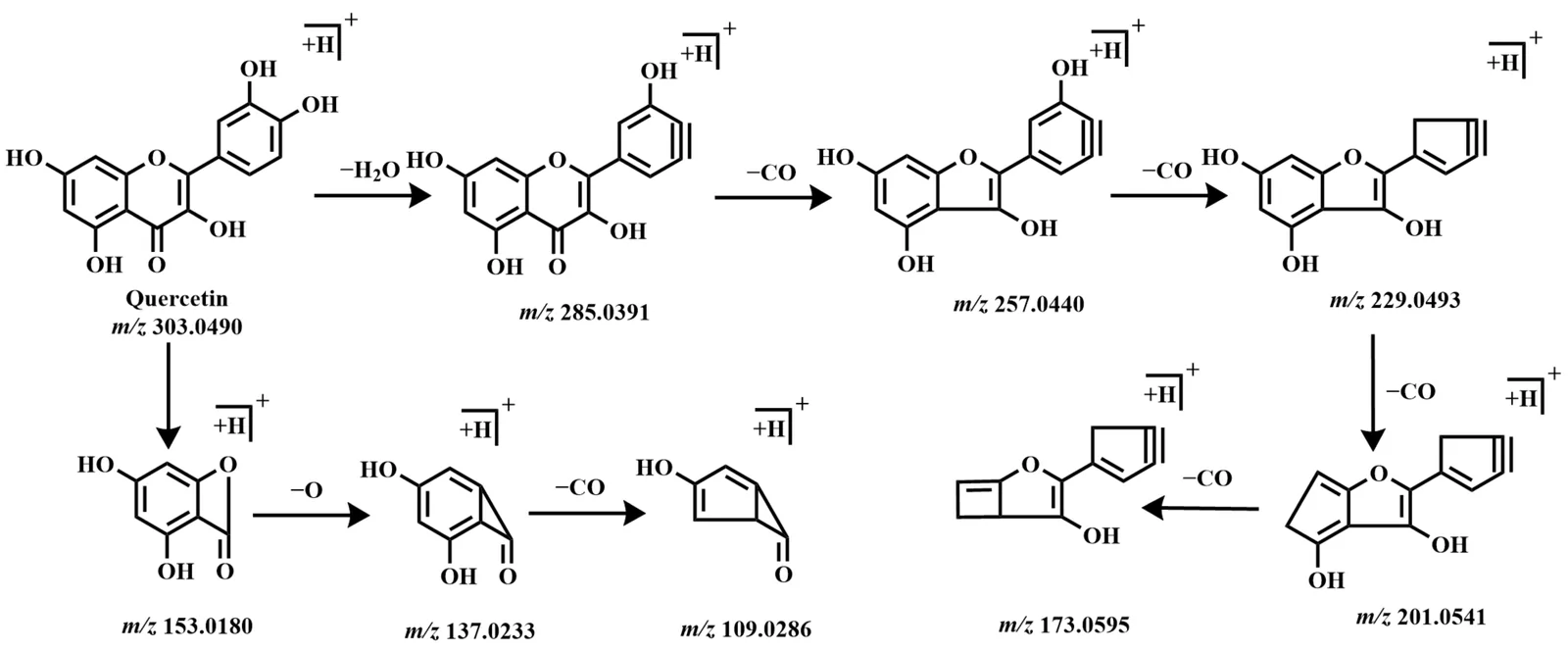
Chemical structure and fragmentation pathways of quercetin.

Compound **3** exhibited a protonated molecular ion [M+H]^+^ at *m*/*z* 287.0543. The precursor ion first lost H_2_O to give a fragment at *m*/*z* 269.0439, followed by successive losses of CO to yield characteristic ions at *m*/*z* 241.0488, *m*/*z* 213.0540, *m*/*z* 185.0593, and *m*/*z* 157.0644. Based on the mass spectrometric fragmentation behavior and comparison with reference data, compound **3** was identified as kaempferol [19]. Compound **4** displayed a protonated molecular ion [M+H]^+^ at *m*/*z* 303.0490, with the molecular formula C_15_H_10_O_7_. In the positive-ion mode, the precursor ion lost H_2_O to give *m*/*z* 285.0391, followed by successive CO losses to yield ions at *m*/*z* 257.0440, *m*/*z* 229.0493, *m*/*z* 201.0541, and *m*/*z* 173.0595. The RDA fragmentation of the C-ring generated a characteristic ion at *m*/*z* 153.0180. Combined with mass spectral database matching and literature comparison [20,21], compound **4** was identified as quercetin, and its proposed fragmentation pathway is shown in Figure 3. Compounds **5** and **6** were identified as morin and myricetin, respectively, by comparison with authentic standards.

#### 3.1.3. Flavanones

**Figure 4:**
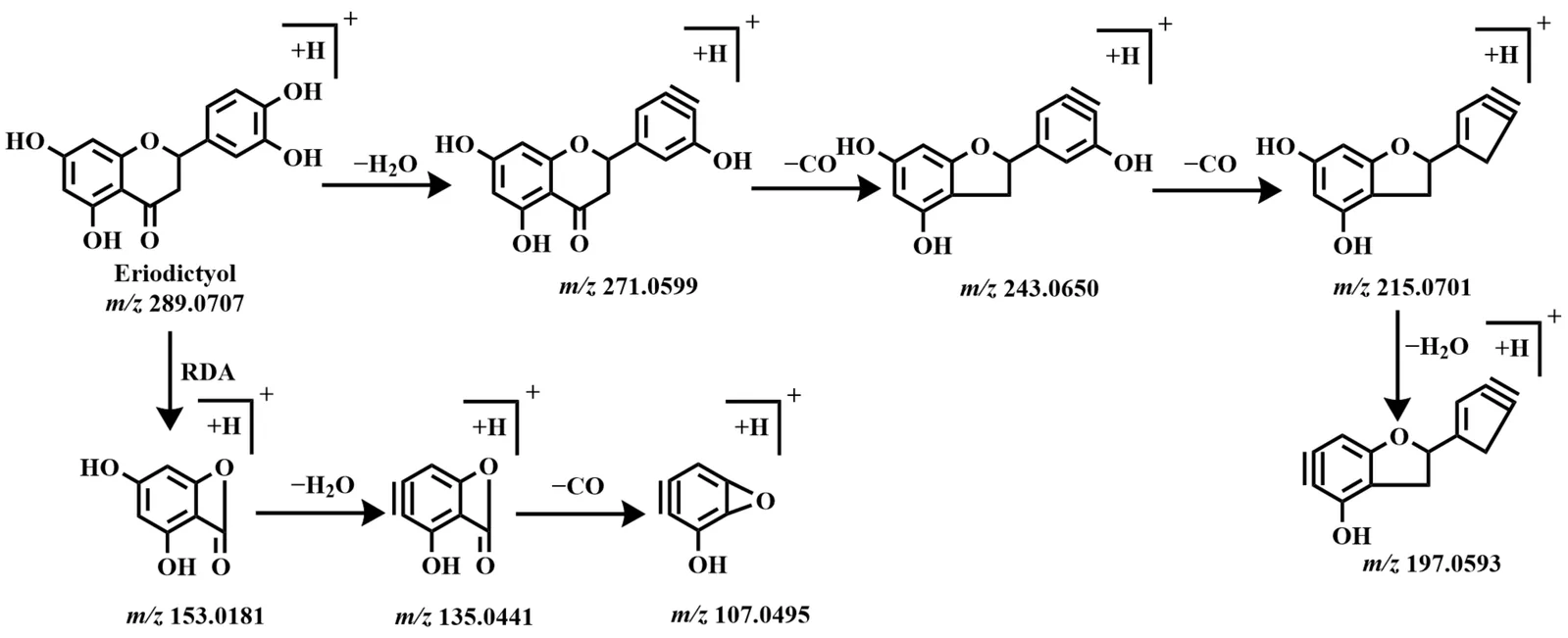
Chemical structure and fragmentation pathways of eriodictyol.

Compounds **7** and **8** were identified as pinocembrin and butin, respectively, based on consistent retention times and MS/MS fragmentation patterns with authentic standards. Compound **9** exhibited a protonated molecular ion [M+H]^+^ at *m*/*z* 273.0756. The precursor ion first lost CO to give *m*/*z* 245.0800, followed by dehydration to yield *m*/*z* 227.0705. Simultaneously, the RDA fragmentation of the C-ring produced a characteristic ion at *m*/*z* 153.0180. Combined with mass spectral database matching, compound **9** was identified as naringenin [12]. Compound **10** displayed a protonated molecular ion [M+H]^+^ at *m*/*z* 289.0707, corresponding to the molecular formula C_15_H_12_O_6_. In the positive-ion mode, the precursor ion first underwent dehydration to produce *m*/*z* 271.0599, followed by successive losses of CO to yield *m*/*z* 243.0650 and *m*/*z* 215.0701, and then further dehydration to generate *m*/*z* 197.0593. The RDA fragmentation generated a characteristic ion at *m*/*z* 153.0181. Combined with database matching, compound **10** was identified as eriodictyol, and its proposed fragmentation pathway is illustrated in Figure 4.

#### 3.1.4. Dihydroflavonols

**Figure 5:**
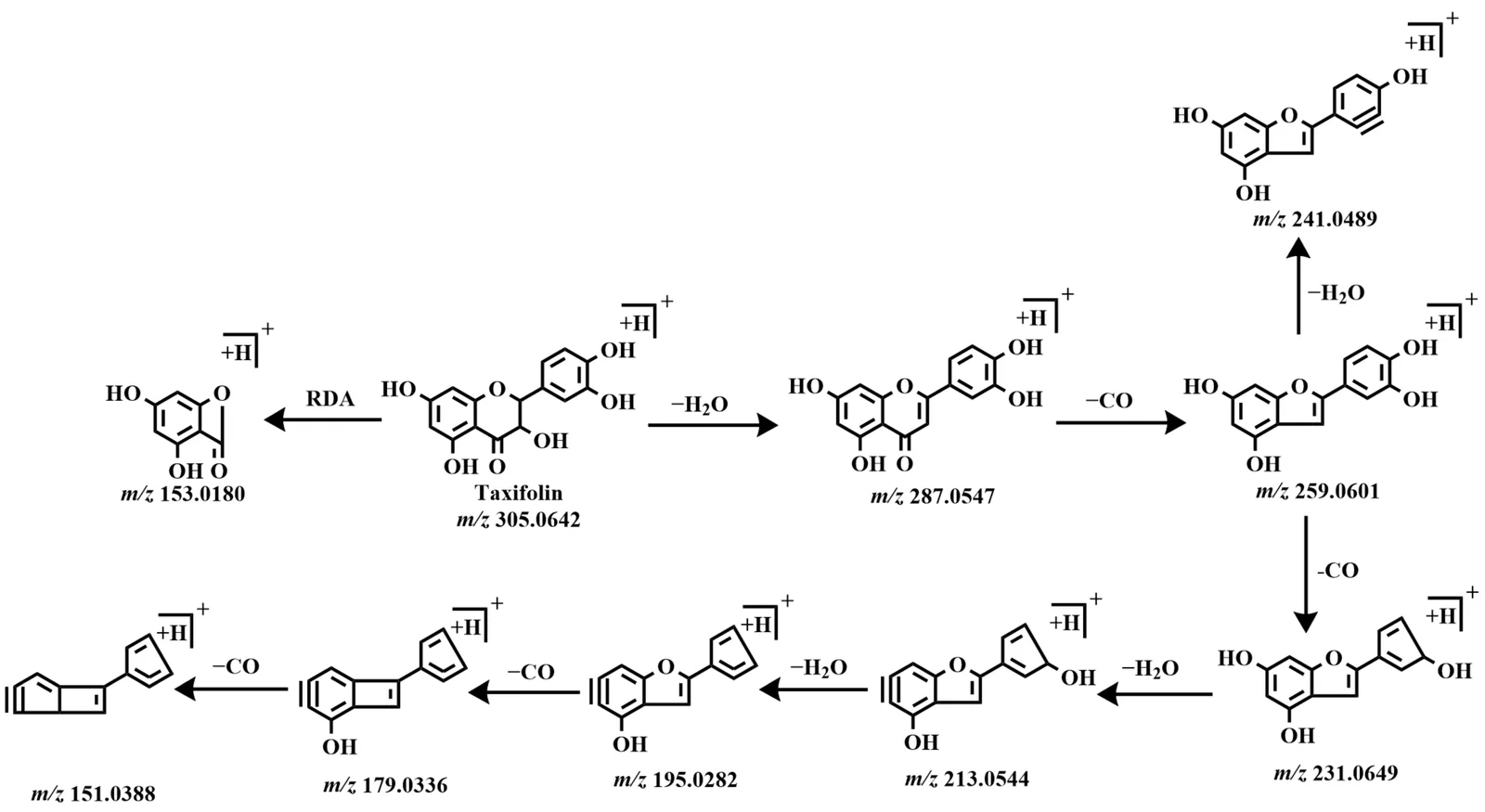
Chemical structure and fragmentation pathways of taxifolin.

Compounds **11**, **12**, and **14** were identified as fustin, dihydrorobinetin, and dihydromyricetin, respectively, by comparison with authentic standards. Compound **13** exhibited a protonated molecular ion [M+H]^+^ at *m*/*z* 305.0642, corresponding to the molecular formula C_15_H_12_O_7_. The major fragment ions included *m*/*z* 287.0547, *m*/*z* 259.0601, *m*/*z* 241.0489, *m*/*z* 231.0649, *m*/*z* 213.0544, *m*/*z* 195.0282, *m*/*z* 185.0592, *m*/*z* 179.0336, *m*/*z* 153.0180, and *m*/*z* 151.0388. Based on mass spectral database matching, compound **13** was identified as taxifolin, and its proposed fragmentation pathway is illustrated in Figure 5.

#### 3.1.5. Flavanols

Compounds **15** and **16** are isomers with the molecular formula C_15_H_14_O_6_. Their retention times and MS/MS fragmentation patterns were consistent with those of authentic standards, and they were identified as (+)-catechin and (−)-epicatechin, respectively. Compound **17** has the molecular formula C_15_H_14_O_7_, with characteristic fragment ions at *m*/*z* 181.0494 and *m*/*z* 139.0393. Combined with mass spectral database matching, it was identified as (+)-gallocatechin. Compound **18** exhibited a protonated molecular ion [M+H]^+^ at *m*/*z* 459.0922, corresponding to the molecular formula C_22_H_18_O_11_. Its characteristic fragment ions included *m*/*z* 289.0700, *m*/*z* 139.0390, *m*/*z* 151.0390, *m*/*z* 121.0285, and *m*/*z* 111.0443. Based on database comparison, it was tentatively identified as epigallocatechin gallate.

#### 3.1.6. Others

Compound **19** displayed a protonated molecular ion [M+H]^+^ at *m*/*z* 255.0644. The precursor ion first lost H_2_O to give *m*/*z* 237.0545, followed by successive losses of CO to yield *m*/*z* 209.0600 and *m*/*z* 181.0645, with additional characteristic ions at *m*/*z* 227.0694 and *m*/*z* 199.0752. Combined with mass spectral database matching, compound **19** was identified as daidzein. Compound **20** exhibited a protonated molecular ion [M+H]^+^ at *m*/*z* 271.0596. The precursor ion successively lost CO to generate *m*/*z* 243.0653 and *m*/*z* 215.0699 and was then dehydrated to give *m*/*z* 197.0594, followed by further CO loss to yield *m*/*z* 169.0645. Other characteristic ions were observed at *m*/*z* 181.0644, *m*/*z* 153.0696, and *m*/*z* 137.0596. Based on database matching, compound **20** was identified as genistein. Compounds **21** to **26** were identified as cyanidin, afzelin, quercitrin, astilbin, isoquercetin, and myricitrin, respectively, by comparison of mass spectral data with authentic standards and in conjunction with literature references.

### 3.2. Content of Characteristic Flavonoid Components in the Xylem of C. hystrix

The commercial value of heartwood is primarily determined by its color and natural durability, both of which are closely associated with the accumulation characteristics of specific flavonoid components. Accordingly, from the 26 flavonoids identified in this study, 10 representative compounds that are highly relevant to heartwood coloration (as condensed tannin precursors), antioxidant activity, and antimicrobial activity were selected. These included the flavanols (+)-catechin and (−)-epicatechin; the dihydroflavonols fustin, dihydrorobinetin, and dihydromyricetin; the flavonols morin and myricetin; the flavanones pinocembrin and butin; and the flavone diosmetin. Using the external standard method, with concentration as the variable and peak area as the response, calibration curves were established for each compound (Table 2) [7,22,23]. The precise quantitative data of these compounds provide direct reference value for evaluating the quality characteristics of *C. hystrix* heartwood and for guiding the extraction of bioactive components from the wood.

Quantitative results (Table 2) showed that the content of (+)-catechin in the heartwood reached 1950.08 mg/kg, approximately 1.9 times that in the sapwood. This exceptionally high accumulation level far exceeded that of all other flavonoid components, suggesting that condensed tannin polymers are likely the major chromogenic substances responsible for the reddish-brown color of *C. hystrix* heartwood. Meanwhile, the contents of dihydromyricetin (233.89 mg/kg) and dihydrorobinetin (145.44 mg/kg) in the heartwood were 17.2 and 4.1 times those in the sapwood, respectively. Not only are these two dihydroflavonols direct precursors of heartwood pigments, but their polyhydroxy structures also confer strong free radical scavenging capacity, which may be associated with the antioxidant durability of the heartwood [24,25]. This marked difference makes morin a potential chemical marker for distinguishing heartwood from sapwood in *C. hystrix* and also indicates that its synthesis and accumulation are highly correlated with the process of heartwood formation.

### 3.3. Pattern Analysis of Heartwood Formation in C. hystrix

**Figure 6:**
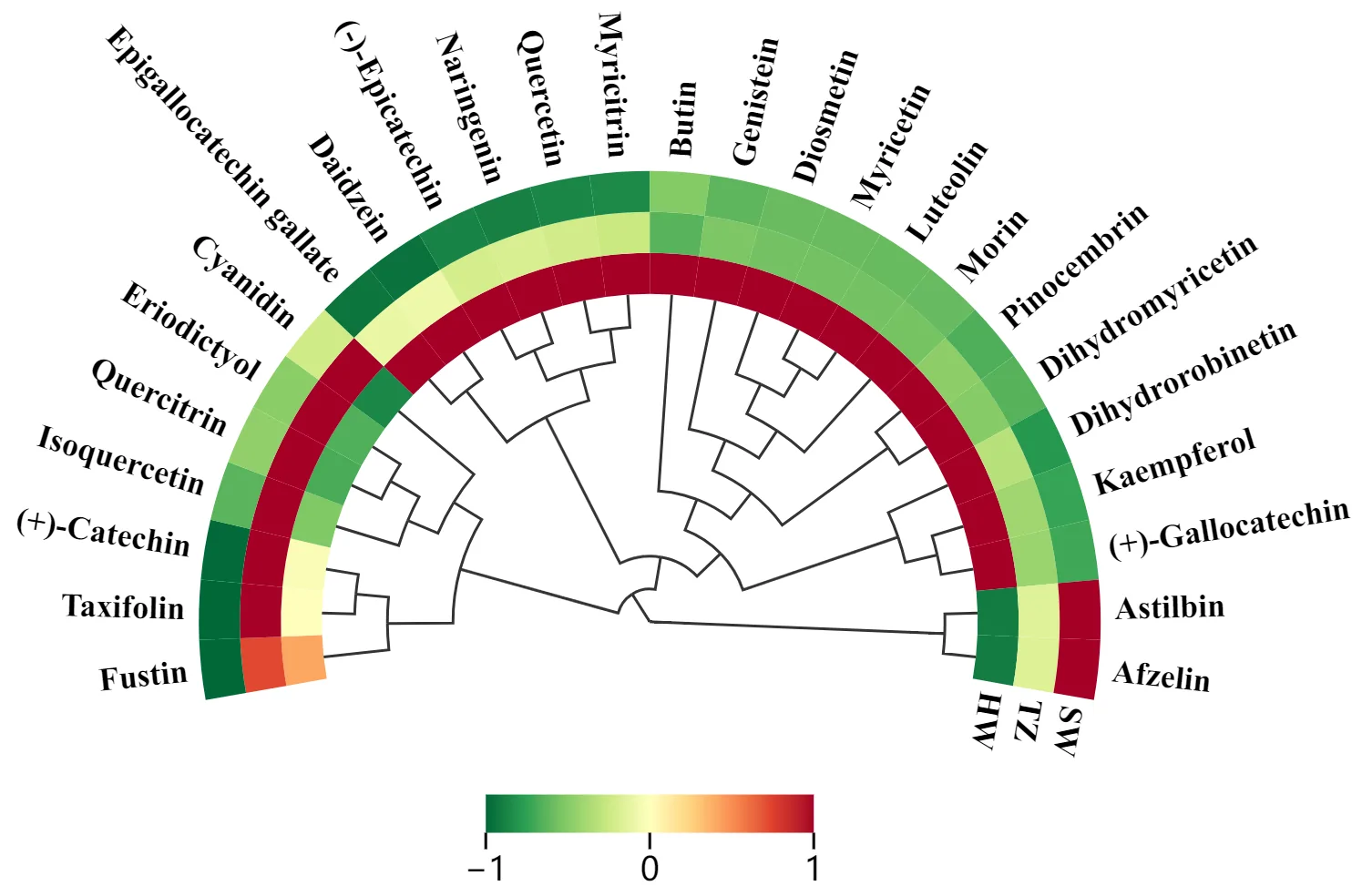
Heatmap analysis of different flavonoid components in the sapwood, transitional zone and heartwood of *Castanopsis hystrix*. Hierarchical clustering analysis was conducted using Euclidean distance as the similarity metric and complete linkage as the agglomeration method, with the data subjected to row-wise Z-score standardization before clustering.

To investigate the spatial dynamics of secondary metabolites during heartwood formation in *C. hystrix*, cluster analysis was performed on the relative abundances of 26 flavonoid compounds in the HW, TZ and SW across the three radial regions, and a circular heatmap was constructed (Figure 6, Table S1). In the heatmap, the color gradient from red to green represents normalized contents from high to low, allowing the visual identification of three distinct accumulation patterns. The first pattern, exemplified by fustin, taxifolin, isoquercetin, (+)-catechin, quercitrin, etc., featured peak contents in the transition zone with relatively low levels in both heartwood and sapwood. This suggests that the transition zone may be the primary site of biosynthesis for these flavonoids, or at least an accumulation site, from which some compounds may subsequently diffuse radially toward the heartwood or sapwood. This pattern is similar to that observed during heartwood formation in black locust [7,8]. Although (+)-catechin follows this accumulation pattern, its content in the transition zone (2964.83 mg/kg) was markedly higher than that in heartwood (1950.08 mg/kg). As a precursor of condensed tannins, (+)-catechin is extensively synthesized in the transition zone and subsequently polymerizes into high-molecular-weight condensed tannins during heartwood formation, which deposit in the cell walls. These polymeric products are poorly soluble in 80% aqueous methanol, leading to a decrease in detectable free monomers in heartwood. The reddish-brown hue of heartwood may therefore primarily arise from the deposition of these polymers. Collectively, content data alone cannot distinguish whether the accumulation in the transition zone reflects in situ synthesis, transport retention, or polymerization conversion; the high levels observed may also result from transient retention of sapwood-synthesized metabolites during centripetal transport or reflect the transitional zone’s role as a metabolic transformation hub.

The second pattern, observed for compounds such as afzelin and astilbin, showed relative enrichment in the sapwood with decreasing contents toward the transition zone and heartwood. A similar pattern occurs in Taxus chinensis, where some secondary metabolites are more abundant in sapwood than in heartwood [13]. It is hypothesized that these compounds may be synthesized in sapwood parenchyma cells but have low radial inward transport efficiency, or they may primarily participate in early defense responses in the sapwood rather than serving as major components for heartwood deposition. The third pattern encompassed the majority of flavonoids, including dihydrorobinetin, kaempferol, pinocembrin, and morin, which exhibited a gradual increase in content from sapwood through transition zone to heartwood. Two possible mechanisms could account for this radial variation. One is that these secondary metabolites are synthesized at low levels in the sapwood, then transported radially inward via ray parenchyma cells and gradually accumulate in the transition zone and heartwood (transport-dominant type). The alternative is that biosynthesis occurs predominantly in the transition zone parenchyma cells, which may be the first to perceive heartwood formation signals and induce the expression of related enzyme systems, with the products subsequently diffusing toward the heartwood and surrounding tissues. The key distinction between these two possibilities lies in the radial location of the peak activity of biosynthetic enzymes and the presence or absence of active transport.

Taken together, the three patterns indicate that the accumulation strategy of flavonoid secondary metabolites during heartwood formation in *C. hystrix* is not governed by a single mechanism; different compounds may follow distinct synthesis–transport pathways. The radial content variations of the metabolites at present only reveal static distribution profiles and cannot directly confirm the sites of biosynthesis. Future studies should systematically measure the radial gradients of key flavonoid biosynthetic enzyme activities, such as phenylalanine ammonia-lyase and chalcone synthase, in the transition zone, sapwood, and heartwood in order to clarify the actual sites of synthesis and transport directions for each compound, thereby providing more robust evidence for the physiological triggers of heartwood formation.

### 3.4. Screening of Heartwood Markers

**Figure 7:**
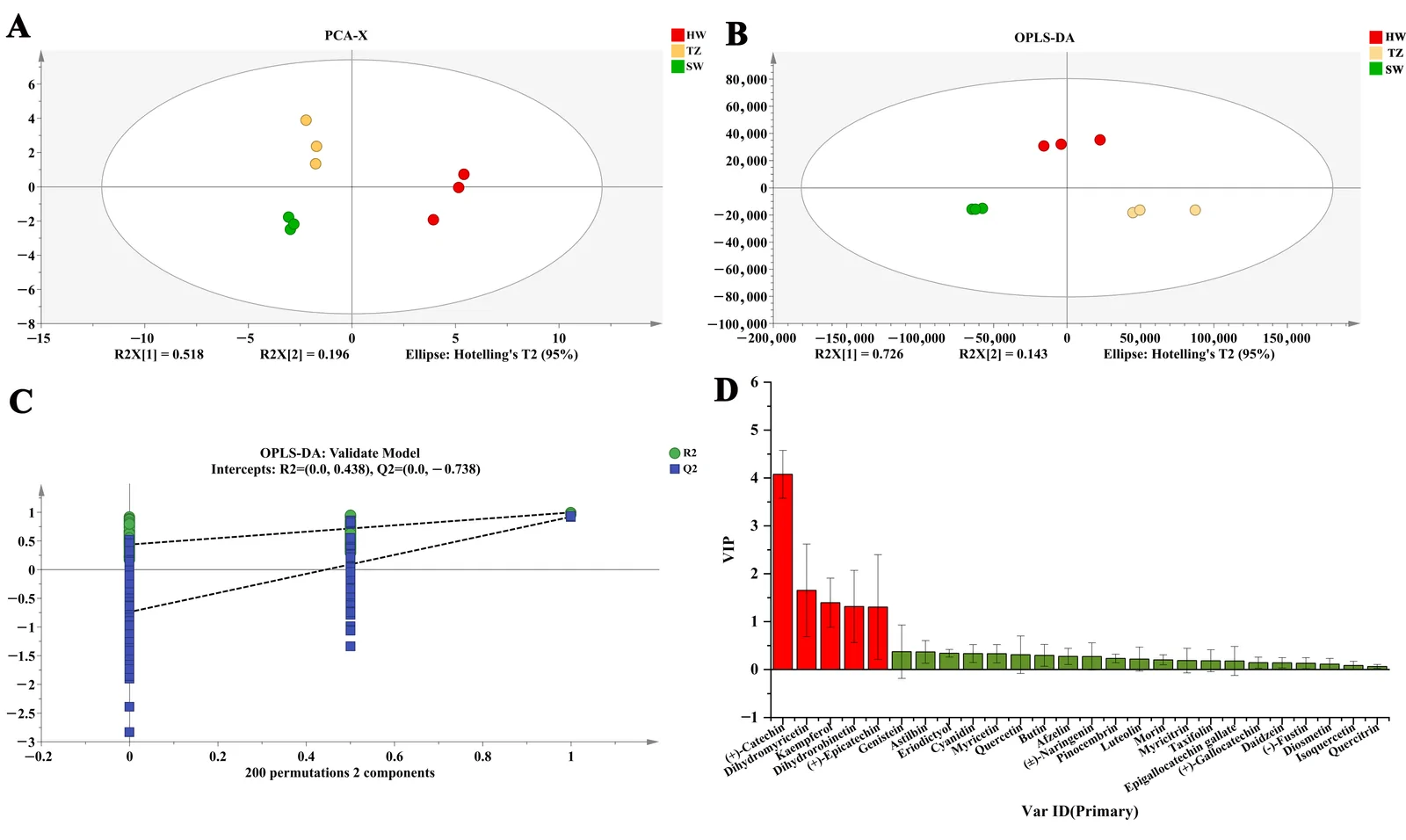
Selection of flavonoid substances based on differences in sapwood (SW), transitional zone (TZ) and heartwood (HW) of *C. hystrix*. (**A**) Principal component score plots of 26 flavonoid components in the sapwood, transitional zone, and heartwood of *Castanopsis hystrix*. (**B**) The OPLS-DA score plots of 26 flavonoid components in the sapwood, transitional zone, and heartwood of *Castanopsis hystrix*. (**C**) Two hundred permutation tests. (**D**) Variable importance in projection (VIP) value plot from the OPLS-DA model, with compounds having VIP > 1 shown in red and those with VIP < 1 shown in green.

To elucidate the tissue specificity of flavonoid accumulation in the xylem of *C. hystrix*, the relative peak areas of 26 flavonoid compounds in sapwood, transition zone, and heartwood were imported into SIMCA 14.1 for principal component analysis (PCA) (Figure 7A). All samples fell within the 95% confidence interval, and the first two principal components cumulatively explained 71.4% of the variance (PC1: 51.8%, PC2: 19.6%). In the score plot, the three groups were completely separated, with the sapwood, transition zone, and heartwood arranged sequentially along PC1, and parallel samples within each group tightly clustered, indicating that the flavonoid accumulation pattern is tissue-specific. This radial variation from sapwood to heartwood is consistent with the process of parenchyma cell programmed cell death and gradual accumulation of secondary metabolites during wood physiological activities. The transition zone samples consistently occupied an intermediate position between sapwood and heartwood in the score plot, suggesting that it is not merely a morphological transition zone but rather a functionally active region of intense metabolic conversion, where flavonoid biosynthetic pathways are progressively activated, thereby laying the precursor foundation for the massive accumulation of characteristic pigments and defensive substances in the heartwood.

Further orthogonal partial least-squares discriminant analysis (OPLS-DA) was performed to distinguish the three groups (Figure 7B). In this model, the predictive component explained 72.6% of the variance in the X matrix, and the orthogonal component explained 14.3%, with clear boundaries among the three groups in the score space. The 200-permutation test (Figure 7C) yielded a negative Q^2^ intercept, confirming that the model was not overfitted. Collectively, these multivariate validation results corroborate the compositional differences observed in the quantitative analyses. Using the criteria of VIP (variable importance in projection) > 1 and fold change (FC, heartwood/sapwood) ≥ 2 (Figure 7D, Table S1), four flavonoid components with the greatest contributions to group discrimination were identified, namely dihydromyricetin, dihydrorobinetin, kaempferol, and (−)-epicatechin. Among the four markers, dihydromyricetin exhibited the highest enrichment fold change (HW/SW = 17.5), suggesting that this polyhydroxy dihydroflavonol is selectively enriched during heartwood formation and may contribute to antioxidant activity and durability [24,25]. Dihydrorobinetin showed a fold change of 4.1, with similarly pronounced accumulation in heartwood. Kaempferol, a known natural antimicrobial component [17], also displayed marked intergroup differences and is thus speculated to enhance the natural decay resistance of heartwood. (−)-Epicatechin, as another key precursor of condensed tannins, is proposed to be synthesized together with (+)-catechin in the transition zone and further polymerized into proanthocyanidins, potentially participating in heartwood coloration.

## 4. Conclusions

In this study, a total of 26 flavonoids were identified from the xylem of *C. hystrix* using UPLC-QE-MS, all of which are reported for the first time in this species. Quantitative analysis revealed that most flavonoid components were enriched in the heartwood, some peaked in the transition zone, and a few were more abundant in the sapwood, reflecting their differential radial distribution across the three xylem regions. PCA and OPLS-DA effectively distinguished the three regions and screened out dihydromyricetin, kaempferol, dihydrorobinetin, and (−)-epicatechin as core differential markers. This set of markers provides quantifiable reference indicators for chemical identification and quality evaluation of *C. hystrix* heartwood. Collectively, these findings offer chemical evidence for elucidating the mechanisms of heartwood formation and lay a material basis for the targeted utilization of bioactive components from this wood species.

## Figures and Tables

**Table 1:** UPLC-QE-MS Analysis of Flavonoids in the Xylem Extract of *Castanopsis hystrix*.

No.	RT	Molecular Formula	Error (ppm)	[M+H]^+^		Main Fragments (*m*/*z*)	Identification
				*m*/*z* Calculated	*m*/*z* Experimental		
Flavones							
**1**	11.707	C_15_H_10_O_6_	−0.08	287.0550	287.0546	269.0439,241.0486,213.0548,153.0182, 137.0232,135.0440	Luteolin ^b^
**2**	12.702	C_16_H_12_O_6_	−0.19	301.0707	301.0704	286.0465,258.0518,153.0182,137.0598	Diosmetin ^a^
Flavonols							
**3**	4.484	C_15_H_10_O_6_	−0.6	287.055	287.0543	269.0439,259.0596,241.0488,213.0540, 185.0593,157.0644	Kaempferol ^b^
**4**	10.138	C_15_H_10_O_7_	−0.3	303.0499	303.0490	285.0391,257.0440,229.0493,201.0541, 173.0595,153.0180,137.0233,109.0286	Quercetin ^b^
**5**	11.236	C_15_H_10_O_7_	−0.19	303.0499	303.0494	285.0384,257.0443,229.0494,201.0543, 173.0586,153.0181,137.0234,135.0440	Morin ^a^
**6**	9.886	C_15_H_10_O_8_	0.27	319.0448	319.0447	301.0338,273.0392,255.0293,245.0439, 217.0495,189.0548,161.0597,153.0182, 137.0234	Myricetin ^a^
Flavanones							
**7**	13.735	C_15_H_12_O_4_	−0.52	257.0808	257.0804	153.0182,103.0546	Pinocembrin ^a^
**8**	9.263	C_15_H_12_O_5_	−0.48	273.0757	273.0754	255.0652,245.0804,137.0234	Butin ^a^
**9**	5.415	C_15_H_12_O_5_	−0.56	273.0758	273.0756	245.0800,227.0705,153.0180,135.0440	Naringenin ^b^
**10**	6.602	C_15_H_12_O_6_	−0.52	289.0707	289.0707	271.0599,243.0650,215.0701,197.0593, 153.0181,135.0441,107.0495	Eriodictyol ^b^
Dihydroflavonols							
**11**	7.236	C_15_H_12_O_6_	−0.27	289.0707	289.0708	271.0595,243.0651,225.0539,215.0701, 197.0595,179.0337,169.0647,137.0233	Fustin ^a^
**12**	5.398	C_15_H_12_O_7_	−0.38	305.0656	305.0655	287.0546,269.0433,259.0597,241.0491, 231.0649,213.0544,195.0285,185.0596	Dihydrorobinetin ^a^
**13**	5.641	C_15_H_12_O_7_	−0.45	305.0656	305.0642	287.0547,259.0601,241.0489,231.0649, 213.0544,195.0282,185.0592,179.0336, 151.0388,153.0180	Taxifolin ^b^
**14**	6.089	C_15_H_12_O_8_	−0.38	321.0605	321.0606	303.0496,275.0546,257.0428,247.0598, 229.0492,219.0288,201.0547,153.0181	Dihydromyricetin ^a^
Flavanols							
**15**	5.158	C_15_H_14_O_6_	−0.89	291.0863	291.0877	153.0558,139.0389,123.0442	(+)-Catechin ^a^
**16**	6.408	C_15_H_14_O_6_	−0.59	291.0863	291.0861	139.0389,123.0442,111.0444,95.0495	(−)-Epicatechin ^a^
**17**	8.525	C_15_H_14_O_7_	−0.28	307.0812	307.0802	181.0494,139.0393	(+)-Gallocatechin ^b^
**18**	6.22	C_22_H_18_O_11_	−0.44	459.0922	459.0922	289.0700,139.0390,151.0390,121.0285, 111.0443	Epigallocatechin gallate ^b^
Others							
**19**	4.527	C_15_H_10_O_4_	−0.59	255.0652	255.0644	237.0545,227.0694,209.0600,199.0752, 181.0645	Daidzein ^b^
**20**	7.061	C_15_H_10_O_5_	−0.78	271.0601	271.0596	243.0653,215.0699,197.0594,169.0645, 181.0644,153.0696,137.0596	Genistein ^b^
**21**	3.629	C_15_H_10_O_6_	−0.34	287.055	287.0554	269.0440,259.0598,241.0491,213.0542, 185.0596,157.0647	Cyanidin ^b^
**22**	6.375	C_21_H_20_O_10_	−0.84	433.1129	433.1125	153.0181,137.0595,125.0233	Afzelin ^b^
**23**	9.638	C_21_H_20_O_11_	0.13	449.1078	449.1079	317.0313,303.0137,287.0548,153.0181, 137.0223	Quercitrin ^b^
**24**	6.374	C_21_H_22_O_11_	0.42	451.1235	451.1753	153.0186,137.0597,119.0491	Astilbin ^b^
**25**	10.072	C_21_H_20_O_12_	1.34	465.1028	465.1034	317.0290,302.0058,285.0028,257.0078, 229.0127,201.0181,173.0234,137.0598, 121.0282	Isoquercetin ^b^
**26**	8.983	C_21_H_20_O_12_	0.13	465.1028	465.1033	319.0443,273.0394,245.0447,217.0497, 153.0182,137.0233	Myricitrin ^b^

Note: ^a^ Compounds assigned to MSI Level 1. ^b^ Compounds assigned to MSI Level 2.

**Table 2:** Content of 10 Flavonoid Components in the Xylem of *Castanopsis hystrix*.

Compounds	Standard Curve	R^2^	Heartwood (mg/kg)	Transition Zone (mg/kg)	Sapwood (mg/kg)
Pinocembrin	y = 88,563x – 922,419	0.998	1.03 ± 0.14 ^a^	0.21 ± 0.12 ^b^	0.11 ± 0 ^b^
Butin	y = 36,708x + 105,661	0.999	5.28 ± 1.53 ^a^	1.44 ± 0.30 ^b^	1.74 ± 0.54 ^b^
Fustin	y = 30,416x – 366,464	0.999	7.75 ± 0.40 ^a^	1.39 ± 0.78 ^b^	1.04 ± 0.31 ^b^
(+)-Catechin	y = 35,676x + 389,456	0.999	1950.08 ± 635.15 ^ab^	2964.83 ± 416.12 ^a^	1023.16 ± 45.79 ^b^
(−)-Epicatechin	y = 30,342x – 323,124	0.999	127.46 ± 38.16 ^a^	58.41 ± 13.14 ^b^	30.81 ± 5.72 ^b^
Morin	y = 38,970x + 100,000	0.999	1.56 ± 0.76 ^a^	0.06 ± 0.01 ^b^	0.02 ± 0.02 ^b^
Dihydrorobinetin	y = 27,267x – 253,894	0.999	145.44 ± 49.30 ^a^	62.2 ± 26.98 ^ab^	35.77 ± 16.68 ^b^
Myricetin	y = 17,102x – 651,267	0.999	10.01 ± 2.55 ^a^	0.76 ± 0.23 ^b^	0.57 ± 0.12 ^b^
Dihydromyricetin	y = 19,526x – 378,539	0.999	233.89 ± 144.96 ^a^	31.8 ± 15.35 ^ab^	13.59 ± 7.07 ^b^
Diosmetin	y = 83,290x – 988,541	0.998	0.36 ± 0.13 ^a^	0.13 ± 0.01 ^b^	0.13 ± 0 ^b^

Note: Different superscript letters (a, b) within the same row indicate significant differences (*p* < 0.05).

## Data Availability

The original contributions presented in the study are included in the article/Supplementary Materials. Further inquiries can be directed to the corresponding authors.

## Supplementary Materials

The following supporting information can be downloaded at: https://www.mdpi.com/article/10.3390/metabo16090655/s1, Table S1: Relative abundances (peak areas) of 26 flavonoid compounds in the sapwood, transitional zone, and heartwood of *Castanopsis hystrix* xylem. Figure S1: Total ion chromatograms of *Castanopsis hystrix* xylem extracts obtained by UPLC-QE-MS in positive ion mode: (A) heartwood, (B) transition zone, and (C) sapwood.

## Abbreviations

The following abbreviations are used in this manuscript:

HW	Heartwood
SW	Sapwood
TZ	Transition Zone
UPLC-QE-MS	Ultra-performance liquid chromatography coupled with Q-Exactive Orbitrap mass spectrometry

## References

[1] Liu X., Li C., Chen S., Wei L., Chen G., Tang J. (2026). Main physical and mechanical properties and their correlations of *Castanopsis hystrix* wood at different tree ages. J. Northwest For. Univ..

[2] Xu B., Zhang F., Pan W., Liu Y. (2013). Genetic diversity and genetic structure in natural populations of *Castanopsis hystrix* from its main distribution in China. Sci. Silvae Sin..

[3] Xue G., Wu J., Zhou B., Zhu X., Zeng J., Ma Y., Wang Y., Jia H. (2023). Effects of shading on the growth and photosynthetic fluorescence characteristics of *Castanopsis hystrix* seedlings of top community-building species in southern subtropical China. Forests.

[4] Meng S., Su Y., Huang A., Sun B. (2023). Radial cracks in *Castanopsis hystrix* wood and its dimensional stability improvement by resin-impregnated modification. Holzforschung.

[5] Liu G., Jia H., Shen W., Xu J. (2022). Comprehensive selection and variation analysis of growth traits and wood color of *Castanopsis hystrix* half-sib families. Pak. J. Bot..

[6] Yang S., Qin F., Wang S., Li X., Zhou Y., Meng S. (2025). Advances in the study of heartwood formation in trees. Life.

[7] Celedon J.M., Bohlmann J. (2018). An extended model of heartwood secondary metabolism informed by functional genomics. Tree Physiol..

[8] Kampe A., Magel E. (2013). New Insights into Heartwood and Heartwood Formation.

[9] Belt T., Keplinger T., Hänninen T., Rautkari L. (2017). Cellular level distributions of Scots pine heartwood and knot heartwood extractives revealed by Raman spectroscopy imaging. Ind. Crops Prod..

[10] Hu W., Nie H., Wang Y., Li N., Di S., Pan Q., Liu J., Han Y. (2022). Tracing the migration and transformation of metabolites in xylem during wood growth by mass spectrometry imaging. Analyst.

[11] Cao S., Deng H., Zhao Y., Zhang Z., Tian Y., Sun Y., Li Y., Zheng H. (2022). Metabolite profiling and transcriptome analysis unveil the mechanisms of red-heart Chinese fir [*Cunninghamia lanceolata* (lamb.) hook] heartwood coloration. Front. Plant Sci..

[12] Ma R., Luo J., Qiao M., Fu Y., Zhu P., Wei P., Liu Z. (2022). Chemical composition of extracts from *Dalbergia odorifera* heartwood and its correlation with color. Ind. Crops Prod..

[13] Shao F., Zhang L., Guo J., Liu X., Ma W., Wilson I.W., Qiu D. (2019). A comparative metabolomics analysis of the components of heartwood and sapwood in *Taxus chinensis* (Pilger) Rehd. Sci. Rep..

[14] Yang X., Yu X., Liu Y., Shi Z., Li L., Xie S., Zhu G., Zhao P. (2021). Comparative metabolomics analysis reveals the color variation between heartwood and sapwood of Chinese fir (*Cunninghamia lanceolata* (lamb.) hook). Ind. Crops Prod..

[15] Rodríguez Anda R., Koch G., Richter H., Fuentes Talavera F.J., Silva Guzmán J.A., Satyanarayana K.G. (2019). Formation of heartwood, chemical composition of extractives and natural durability of plantation-grown teak wood from Mexico. Holzforschung.

[16] Morris H., Hietala A.M., Jansen S., Ribera J., Rosner S., Salmeia K.A., Schwarze F.W.M.R. (2020). Using the CODIT model to explain secondary metabolites of xylem in defence systems of temperate trees against decay fungi. Ann. Bot..

[17] Cai M., Lv H., Cao C., Zhang L., Cao R., Xu B. (2019). Evaluation of antimicrobial activity of *Pterocarpus* extracts. Ind. Crops Prod..

[18] Sumner L.W., Amberg A., Barrett D., Beale M.H., Beger R., Daykin C.A., Fan T.W.M., Fiehn O., Goodacre R., Griffin J.L. (2007). Proposed minimum reporting standards for chemical analysis. Metabolomics.

[19] Jiang C., Gates P.J. (2024). Systematic characterisation of the fragmentation of flavonoids using high-resolution accurate mass electrospray tandem mass spectrometry. Molecules.

[20] Tsimogiannis D., Samiotaki M., Panayotou G., Oreopoulou V. (2007). Characterization of flavonoid subgroups and hydroxy substitution by HPLC-MS/MS. Molecules.

[21] Tan L., Zhang D., Wang G., Yu B., Zhao S., Wang J., Yao L., Cao W. (2016). Comparative analyses of flavonoids compositions and antioxidant activities of hawk tea from six botanical origins. Ind. Crops Prod..

[22] Ninh T.S. (2017). A review on the medicinal plant *Dalbergia odorifera* species: Phytochemistry and biological activity. Evid.-Based Complement Altern. Med..

[23] Sanz M., Fernández De Simón B., Esteruelas E., Muñoz Á.M., Cadahía E., Hernández T., Estrella I., Pinto E. (2011). Effect of toasting intensity at cooperage on phenolic compounds in acacia (*Robinia pseudoacacia*) heartwood. J. Agric. Food Chem..

[24] Li C., Huang K., Li K. (2025). Combined analysis of the metabolome and transcriptome reveals the mechanism of red heart Chinese fir heartwood formation. Sci. Rep..

[25] Vek V., Poljanšek I., Oven P. (2019). Efficiency of three conventional methods for extraction of dihydrorobinetin and robinetin from wood of black locust. Eur. J. Wood Wood Prod..

