# Supplementary material for: Identification of Flavonoids in the Xylem of Castanopsis hystrix and Their Content Variation Patterns During Heartwood Formation
Source: Metabolites. 2026 Sep 8;16(9):655. doi: 10.3390/metabo16090655 (PMC13609397; doi:10.3390/metabo16090655)
Supplement: Supplementary file 1 [file metabolites-16-00655-s001.zip › Fiure S1.pdf]

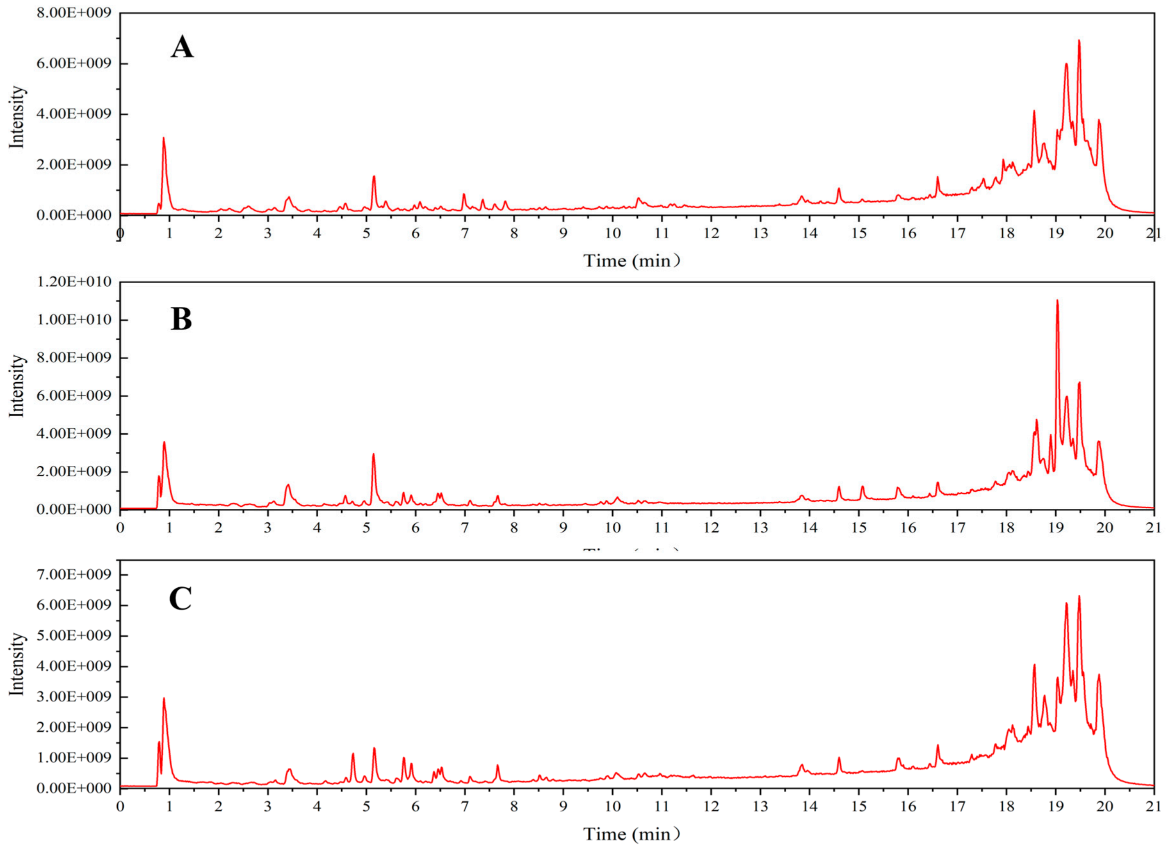

Figure S1: Total ion chromatograms of *Castanopsis hystrix* xylem extracts obtained by UPLC-QE-MS in positive ion mode: (A) heartwood, (B) transition zone, and (C) sapwood.
